# Predicting response to radiotherapy of intracranial metastases with hyperpolarized $^{13}$C MRI

**DOI:** 10.1007/s11060-021-03725-7

**Published:** 2021-03-19

**Authors:** Casey Y. Lee, Hany Soliman, Nadia D. Bragagnolo, Arjun Sahgal, Benjamin J. Geraghty, Albert P. Chen, Ruby Endre, William J. Perks, Jay S. Detsky, Eric Leung, Michael Chan, Chris Heyn, Charles H. Cunningham

**Affiliations:** 1 Department of Medical Biophysics, University of Toronto, Toronto, ON Canada; 2 Physical Sciences, Sunnybrook Research Institute, M7-613, 2075 Bayview Avenue, Toronto, ON M4N 3M5 Canada; 3 Radiation Oncology, Sunnybrook Health Sciences Centre, Toronto, ON Canada; 4 GE Healthcare, Toronto, Ontario Canada; 5 Pharmacy, Sunnybrook Health Sciences Centre, Toronto, ON Canada; 6 Radiology, Trillium Health Partners, Mississauga, ON Canada; 7 Radiology, Sunnybrook Health Sciences Centre, Toronto, ON Canada

**Keywords:** Lactate, Hyperpolarized 13C MRI, Metabolism, Intracranial metastases, SRS

## Abstract

**Background:**

Stereotactic radiosurgery (SRS) is used to manage intracranial metastases in a significant fraction of patients. Local progression after SRS can often only be detected with increased volume of enhancement on serial MRI scans which may lag true progression by weeks or months.

**Methods:**

Patients with intracranial metastases (N = 11) were scanned using hyperpolarized $^{13}$C MRI prior to treatment with stereotactic radiosurgery (SRS). The status of each lesion was then recorded at six months post-treatment follow-up (or at the time of death).

**Results:**

The positive predictive value of $^{13}$C-lactate signal, measured pre-treatment, for prediction of progression of intracranial metastases at six months post-treatment with SRS was 0.8 $p < 0.05$, and the AUC from an ROC analysis was 0.77 $p < 0.05$. The distribution of $^{13}$C-lactate *z*-scores was different for intracranial metastases from different primary cancer types (F = 2.46, $p = 0.1$).

**Conclusions:**

Hyperpolarized $^{13}$C imaging has potential as a method for improving outcomes for patients with intracranial metastases, by identifying patients at high risk of treatment failure with SRS and considering other therapeutic options such as surgery.

**Supplementary Information:**

The online version contains supplementary material available at 10.1007/s11060-021-03725-7.

## Importance of study

Stereotactic radiosurgery (SRS) is used to manage intracranial metastases in a significant fraction of patients. Local progression after SRS can often only be detected with increased volume of enhancement on serial MRI scans which may lag true progression by weeks or months. In this work, a safe and non-invasive method that has already been translated to patient studies is shown to be a promising new predictor of treatment failure. Predictive tools for radiation resistance prior to SRS may help identify patients better suited for upfront surgical resection or SRS dose escalation. Since the method is integrated with MRI, it can be added into the pre-treatment MRI used for radiation treatment planning with minimal change to patient workflow. The results are actionable and have the potential to improve outcomes, as the tumour lactate measurement is available prior to treatment with SRS.

## Introduction

Upwards of 20% of cancer patients will develop brain metastases during the course of their illness, many of which become symptomatic [1–4]. The most common primary tumours responsible for brain metastases include lung cancer, melanoma, renal cancer, breast cancer, and colorectal cancer. The management of brain metastases has become more complex and may involve surgery, stereotactic radiosurgery (SRS), whole brain radiotherapy (WBRT), systemic therapy or best supportive care [5].

Over the past several decades, with improved imaging and radiation therapy technology, stereotactic radiosurgery (SRS) has cemented a role in the treatment of a significant proportion of patients with brain metastases. SRS has been shown to improve survival in patients with brain metastases over WBRT alone [6] and avoids the the neurocognitive side effects of WBRT [7]. However, local control of brain metastases with SRS decreases with increasing tumour diameter as the dose is dialed back to reduce the risk of radiation injury [8, 9]. Local progression can often only be detected with increased volume of enhancement on serial MRI scans which may lag true progression by weeks or months [10, 11]. Therefore predictive tools for radiation resistance prior to SRS may help identify patients better suited for dose escalation or even upfront surgical resection. If the risk of treatment failure could be better assessed beforehand, some of these patients might be reclassified as candidates for surgery, improving outcomes.

A proposed indicator of SRS treatment failure is tumour lactate accumulation. The impact of lactate accumulation on radiosensitivity was only recognized relatively recently [12], but is now well established. Lactate accumulates in malignant tumours through a number of mechanisms, including hypoxia-inducible factor 1 (HIF-1) mediated reprogramming. It has been hypothesized that lactate affects radioresistance by antioxidant properties, inducing angiogenesis, mediating resistance to apoptosis, as well as by stabilizing HIF-1$\alpha$ and perpetuating the activation of HIF-1 *independent of* hypoxia [13]. Tumour lactate levels were shown to be inversely correlated with overall and disease-free patient survival in cervical cancer, head and neck squamous cell carcinoma, and glioblastoma in humans [14–17]. However, these findings were all based on lactate measurements in biopsy or surgical tumour samples, which are not available pre-treatment for intracranial metastases.

Hyperpolarized (HP) $^{13}$C MRI is an new imaging approach that has the potential to predict radiation treatment failure by probing lactate metabolism in vivo. This technique uses a non-radioactive labelled metabolite, [1-$^{13}$C]pyruvate, as a contrast agent. Prior to imaging, the signal of [1-$^{13}$C]pyruvate is amplified by approximately 10,000-fold, or “hyperpolarized”, via dynamic nuclear polarization [18]. While the amplified signal is short-lived (exponential decay constant of $\sim$ 40s in vivo), images of [1-$^{13}$C]pyruvate and its metabolic products [1-$^{13}$C]lactate and $^{13}$C-bicarbonate can be obtained within a 1-minute time window after injection. Clinical applications of HP $^{13}$C MRI have been explored in recent years, starting with the first in-human imaging of prostate cancer patients in 2013 [19]. The application of HP $^{13}$C MRI in patients with brain tumours and metastases is currently an active area of investigation [20–22].

In this work, patients (N=11) with one or more newly developed intracranial metastases were scanned using hyperpolarized $^{13}$C MRI prior to treatment with SRS. The mean $^{13}$C-lactate signal from each lesion was normalized using the consistent pattern of $^{13}$C-lactate in the brain parenchyma. The status of each lesion was then recorded at the six months post-treatment follow-up (or at the time of death). This enabled estimation of the positive predictive value of pre-treatment [1-$^{13}$C]lactate signals and receiver operating characteristic (ROC) curve prediction of lesion progression at six months post-treatment with SRS.

## Materials and methods

Written informed consent was obtained from patients with intracranial metastases (N = 11) prior to study participation under a protocol approved by the institutional Research Ethics Board and by Health Canada as a Clinical Trial Application. All participants had one or more newly developed intracranial metastasis and were scheduled for SRS immediately following the $^{13}$C imaging study.

Prior to being positioned head-first in a General Electric (GE) MR750 3.0T MRI scanner (GE Healthcare, Waukesha, WI), a 22-gauge intravenous catheter was inserted into each patient’s forearm. The patient’s head was then secured in the support for the head coil base for a standard 8-channel neurovascular receive array (Invivo Inc.). This support could be docked with either the 8-channel $^1$H array or a home-built single-tuned $^{13}$C birdcage coil without moving the patient’s head during the study. At the beginning of each exam, localizer images and a reference scan to be used during the $^{13}$C image reconstruction [23] were acquired using the scanner’s built-in body coil. The $^{13}$C birdcage coil was then put in place.

Each subject was injected with a 0.43 mL/kg dose of 250 mM [1-$^{13}$C]pyruvate via an intravenous injection at 4 mL per second using a MEDRAD Spectris solaris injector (Bayer). The doses were prepared within a sterile fluid path and hyperpolarized in a GE SPINLab polarizer equipped with a quality control module. The $^{13}$C image acquisition was initiated upon the completion of a saline flush. All patients tolerated the [1-$^{13}$C]pyruvate injection without any adverse events.

A three-dimensional (3D) spectrally-selective echo-planar imaging (SS-EPI) [23, 24] pulse sequence was used to acquire time-resolved full brain data from [1-$^{13}$C]lactate, [1-$^{13}$C]bicarbonate and [1-$^{13}$C]pyruvate signals (5-s temporal resolution; 12 time points; 1.5-cm isotropic spatial resolution with a 24 $\times$ 24 $\times$ 36 cm$^3$ field of view).

Following the $^{13}$C image acquisition, the $^{13}$C head coil was replaced with an 8-channel $^1$H neurovascular array (Invivo Inc.) for a standard suite of anatomical brain image acquisitions. T$_1$-weighted images were acquired using 3D fast spoiled GRE (axial prescription, FOV 25.6 $\times$ 25.6 cm$^2$, 1-mm isotropic resolution, TR 7.6 ms, TE 2.9 ms, flip angle 11$^\circ$). Gadolinium enhanced T$_1$-weighted images were acquired 2 min after administering a gadolinium dose of 0.1 mmol/kg via hand injection in a subset of patients that required these images for radiation treatment planning. T$_2$-weighted images were acquired using T$_2$-FLAIR (axial, FOV 22 $\times$ 22 cm$^2$, in-plane resolution 0.6875 $\times$ 0.982 mm$^2$, 3-mm slice thickness, TR/TE 8000/120 ms, flip angle 111$^\circ$).

All $^{13}$C image reconstruction was performed offline using MATLAB R2018b (The MathWorks Inc., MA, Natick, Massachusetts). Data-driven geometric distortion artifact correction was performed as in [23]. The time-resolved images were summed to compute the area-under-the-curve (AUC) for each metabolite, which were then stored as final $^{13}$C images.

The structural parcellation of metabolite signals within the brain was performed using BrainParser, which uses T$_1$-weighted images to parcellate the brain into the 56 structural regions contained in the LPBA40 atlas [25]. For each subject the mean $\mu$ and standard deviation $\sigma$ of the $^{13}$C-lactate signals across the 56 atlas regions were computed. Then the subject’s lactate value for each region, $x_i$, was normalized by converting it to a *z*-score, $z_i$, as in [26]:

1 $$\begin{aligned} z_i = (x_i - \mu ) / \sigma \end{aligned}$$

A region of interest (ROI) for each metastasis was manually contoured onto each slice of the post-gadolinium T$_1$-weighted images, if available, by a radiation oncologist. In cases where post-gadolinium T$_1$-weighted images were not acquired, manual contouring was performed on the non-contrast T$_1$-weighted images or the T$_2$-weighted images. The mean $^{13}$C metabolite signal from each lesion, $x_{lesion}$, was converted to a *z*-score, $z_{lesion}$, using the mean and standard deviation of the $^{13}$C-lactate signal across anatomical brain regions, as follows:

2 $$\begin{aligned} z_{lesion} = (x_{lesion} - \mu ) / \sigma \end{aligned}$$

where $\mu$ and $\sigma$ are as defined above, based on the 56 brain atlas regions for that patient. The normalization of the lactate signal by converting to a *z*-score is needed because the level of polarization of the $^{13}$C-substrate at the time it is detected in each subject, which determines the signal strength, varies over a wide range and this would otherwise confound the measurement.

Treated brain metastases were evaluated at the 6 month follow-up or last follow up prior to death. Tumour response was evaluated according to the RANO brain metastases criteria [11]. No cases of radiation necrosis were seen. Lesion locations and lactate *z*-scores are summarized in Table 1 (Supplementary).

## Results

**Fig. 1:**
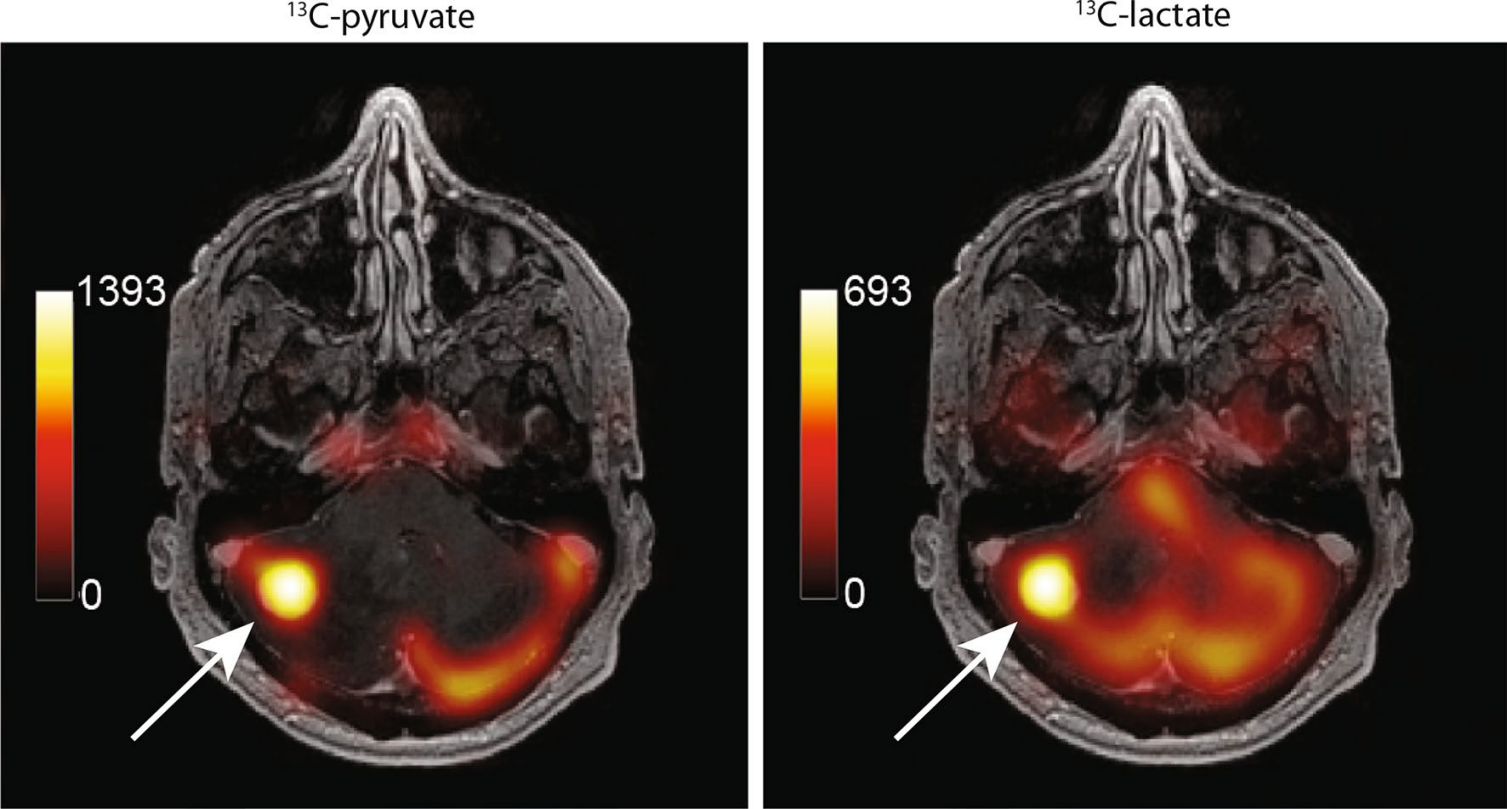
Example $^{13}$C-metabolite images of an intracranial metastasis (arrows) in a renal cell carinoma (RCC) patient. The metabolite signals are displayed as colour overlays on the corresponding greyscale T1-weighted anatomical images, and were computed by summing 12 time-points collected over a 60 s acquisition window. The treatment of this high-lactate lesion with SRS failed, with local progression prior to the 6-month followup

**Fig. 2:**
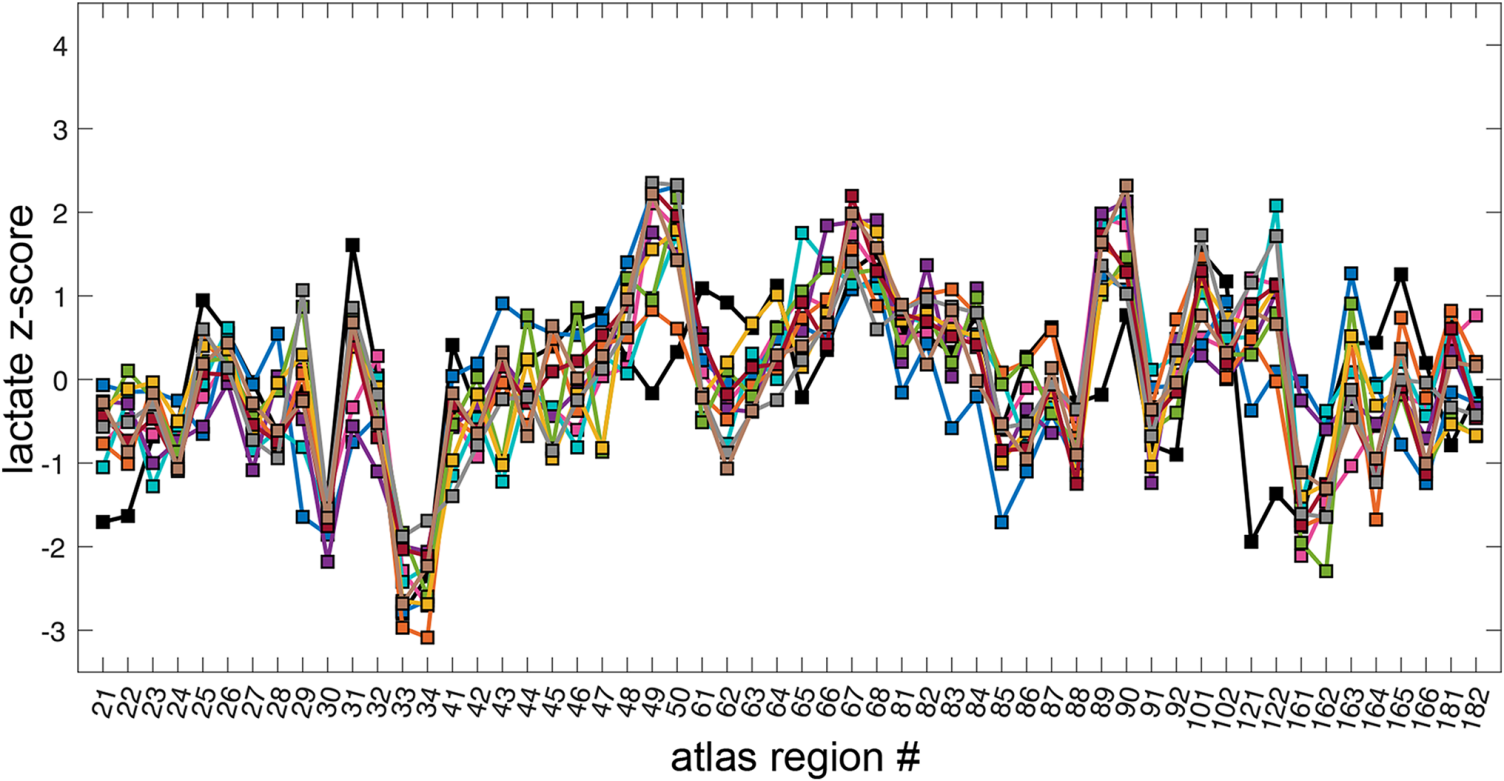
Lactate *z*-scores from 56 LPBA40 atlas regions for 11 patients. The atlas regions are indicated with LPBA40 atlas region numbers on the horizontal. The range of region *z*-scores was consistent across subjects

Figure 1 shows an example of $^{13}$C-lactate and $^{13}$C-pyruvate images from one subject. In Fig. 2, the $^{13}$C-lactate pattern across cortical regions, expressed as a plot of *z*-score vs. atlas region, appeared similar to the pattern observed in healthy participants, but with weaker concordance across patients (Kendall’s *W* = 0.70) as compared to healthy participants (*W* = 0.83) [26]. Despite the moderate reduction in concordance, the range of lactate *z*-scores from the 56 atlas regions spanned roughly the same range in all subjects, from − 3 to 2, which is similar to the range seen in healthy volunteers [26] and sufficiently consistent across patients to justify the tumour $^{13}$-lactate normalization method of Eq. (2).

**Fig. 3:**
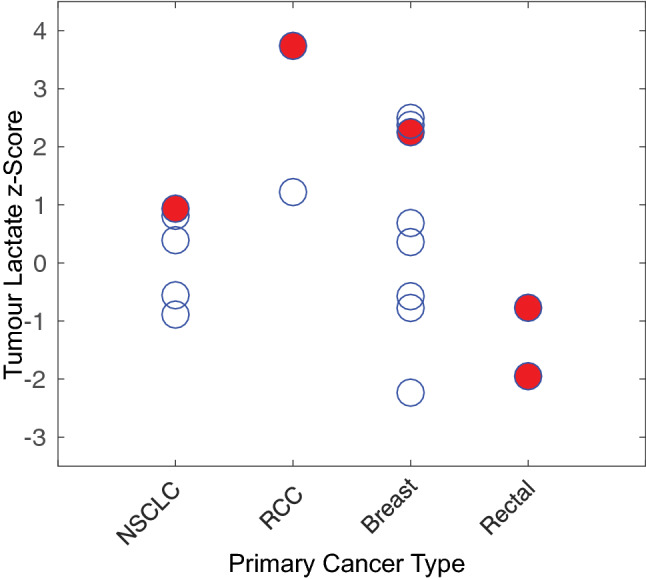
Lactate *z*-scores of newly developed lesions by primary tumour type. The red, solid circles show lesions that progressed, while the other open circles are stable/responding lesions. Note the apparent relationship between the higher lactate *z*-scores for each primary cancer type and progression

The metastatic lesions displayed a broad range of lactate *z*-scores, from − 2.23 to 3.74, as shown in Fig. 3. A different range of lactate *z*-scores was observed for each of the four primary tumour types: non-small cell lung carcinoma (NSCLC, N = 4), renal cell carcinoma (RCC, N = 2), breast cancer (N = 4) and colorerectal cancer (N = 1). To test whether this apparent difference was statistically significant, a 1-way ANOVA was used with the primary cancer type as a categorical variable and the lactate *z*-scores as the dependent variable, giving F = 2.46 and $p = 0.1$.

**Fig. 4:**
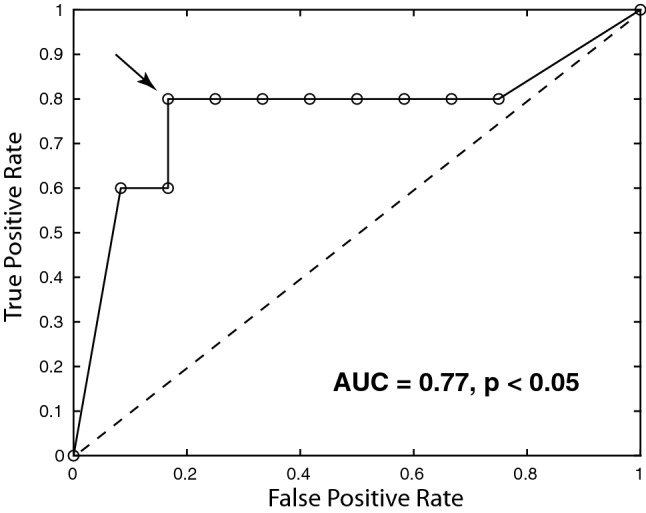
Receiver operating characteristic (ROC) curve for pre-treatment $^{13}$C-lactate scores as a predictor of treatment failure at 6-months post-treatment with SRS. The optimal threshold is indicated with the arrow, giving a true-positive rate of 0.8, a false-positive rate of 0.2 and positive-predictive value of 0.8 ($p < 0.05$)

Based on this result and the observation that the highest lactate *z*-scores from each primary cancer type appeared to be associated with treatment failure (red circles in Fig. 3), the set of lactate *z*-scores for each primary cancer type was rescaled to a prediction score, ranging from 0 to 1. This allowed the prediction score values from all tumour types to be included in the same ROC analysis, resulting in the ROC curve shown in Fig. 4. The area-under-the-curve (AUC) was 0.77 with an optimal threshold giving a true positive rate of 0.8, a false positive rate of 0.2 and a positive predictive value of 0.8. Based on the equivalency relationship between the AUC and the parameter U derived in the Mann–Whitney U test, as described in [27], the statistical significance of this ROC result was calculated to be $p < 0.05$.

## Discussion

The performance of $^{13}$C-lactate as a predictor of treatment failure was superior to other markers previously investigated for this purpose, such as the apparent diffusion coefficient (ADC) [28] measured *after* SRS. ROC analysis on the prediction of progression using ADC values at 1 week and 1 month post-radiation gave an AUC of 0.704 and 0.748, respectively. Furthermore, the AUC of $^{13}$C-lactate would likely improve if ROC analysis was performed on each primary cancer type separately. A limitation of the present study was that lesions from different primary cancers were pooled in the analysis, requiring rescaling to equalize the range of values for each primary cancer. In the future, a sufficient number of patients will be scanned from each primary cancer group so that ROC analysis can be performed separately.

A brain parcellation method was used in this study to normalize $^{13}$C signals from metastatic lesions using the $^{13}$C signals from other brain regions for reference. This normalization method, based on comparison to the consistent background metabolite pattern in the brain, is a key element of the analysis as it allows for a $^{13}$C-lactate measurement of each lesion computed from just the $^{13}$C-lactate images alone. This is important because elevated $^{13}$C-lactate signal has been shown to occur alongside elevated $^{13}$C-pyruvate (substrate) signal under several common conditions such as high tumour vascularity and elevated expression of MCT1 transporters [29]. The conventional analysis methods found in the literature, which involve computation of a lactate-to-pyruvate ratio or fitting a first-order rate constant to time-resolved data, both inherently reduce the number that results from the high-lactate/high-pyruvate condition. This condition, in fact, is likely a highly malignant phenotype. One of the lesions that progressed in the preliminary study fell into this category (the highest $^{13}$C-lactate *z*-score in Fig. 3).

There were several limitations to this study. The cohort of patients was small and somewhat heterogeneous. However, the small number of patients reflects the current expense and logistical challenges associated with hyperpolarized $^{13}$C MRI, which is still in an early trial phase. Lesions from all patients eligible for SRS were included in this preliminary study to test feasibility and enable design of future trials. However, based on these promising results, the next step will be to acquire data from larger, groups of patients grouped by primary cancer.

Another limitation of this study was the coarse spatial resolution used for the $^{13}$C imaging (1.5-cm isotropic). This may have led to contamination of the tumour $^{13}$C-lactate signal from the surrounding tissues, an effect that would be worse for smaller lesions. Thus, the positive predictive value of this method may improve with advances in the data acquisition method enabling higher spatial resolution.

Three lesions across two patients were treated with SRS prior to $^{13}$C imaging. The lactate *z*-scores from these lesions were − 1.13, − 1.29 and 1.04. Future studies should investigate whether the observed *z*-scores in previously treated lesions reflect treatment-related metabolic changes or are consistent with the $^{13}$C-lactate signal levels observed in healthy brain tissues.

## Conclusions

The positive predictive value of tumour $^{13}$C-lactate signals, measured pre-treatment, for the prediction of progression of intracranial metastases at six months post-treatment with SRS was 0.8 $p < 0.05$, and the AUC from an ROC analysis was 0.77 $p < 0.05$. The distribution of $^{13}$C-lactate *z*-scores varied among intracranial metastases from different primary cancer types (F = 2.46, $p = 0.1$). Hyperpolarized $^{13}$C imaging has thus shown potential as a method for improving outcomes in patients with intracranial metastases, by identifying patients at high risk of treatment failure with SRS and who may benefit from other therapeutic options such as surgery.

## Supplementary Information

Below is the link to the electronic supplementary material.

Supplementary information 1 (PDF 751 kb)

## Abbreviations

DE-EPI: Dual-echo echo-planar imaging
HP: Hyperpolarized
LPBA40: LONI Pipeline Brain Atlas 40
NSLSC: Non-small cell lung cancer
RCC: Renal cell carcinoma
ROC: Receiver operating characteristic
*W*: Kendall’s coefficient of concordance
