# Supplementary material for: Predicting response to radiotherapy of intracranial metastases with hyperpolarized 13C MRI
Source: J Neurooncol. 2021 Mar 19;152(3):551–7. doi: 10.1007/s11060-021-03725-7 (PMC8084843; doi:10.1007/s11060-021-03725-7)
Supplement: Supplementary file 1 — Supplementary information 1 (PDF 751 kb) [file 11060_2021_3725_MOESM1_ESM.pdf]

ID#	Age / Gender	Primary	Location	Lac z-score	Status	Dose (Gy)	Fracs	Other organs involved	# Previous systemic therapies
11	75/M	NSCLC	brainstem	-0.89	stable	15	1	lung, brain	0
12	66/M	RCC	R cerebellum	3.74	progressed	18	1	lung	2
20	70/F	NSCLC	L occipital lobe	0.39	responding	20	1	lung, lymph nodes	2
24	57/F	Breast	R parietal	2.50	responding	27.5	5	lymph nodes	2
			R temporal	-0.78	stable				
			L frontal	-1.13	pre-treated				
27	46/F	NSCLC	R frontal	0.81	responding	27.5	5	lung	1
			L frontal	-0.56	responding				
34	60/M	NSCLC	L cerebellum	0.94	progressed	24	3	lung, bone	1
36	58/M	Rectal	L cerebellum	-0.77	progressed	25	5	lung, liver, lymph nodes	3
			R cerebellum	-1.95	progressed				
37	45/M	RCC	L cerebellum	1.22	stable	25	5	kidney, liver, lung, lymph nodes	3
			R cerebellum	-1.29	pre-treated				
			L occipital	1.04	pre-treated				
39	40/F	Breast	R occipital	0.69	responding	25	5		2
			M cerebellum	2.25	progressed				
40	58/F	Breast	L occipital	-2.23	responding	18	1	lung, bone	3
47	55/F	Breast	R occipital	2.38	responding	30	5	liver, lymph nodes	5
			R frontal	-0.57	responding				
			L frontal	0.36	responding				

Table 1: (Supplementary) Summary of patients and lesions data. ID# indicates the assigned subject identifier, location is the lesion location in the brain, status is the clinical status of the lesion at six months post-SRS or death, Fracs is the number of fractions, Previous systemic therapies is the number of lines of systemic therapy prior to brain metastases. Abbreviations: NSCLC: non-small cell lung cancer, RCC: renal cell carcinoma, L: left, R: right, M: medial.
